# Supplementary material for: Therapeutic monitoring of adalimumab at non-trough levels in patients with inflammatory bowel disease
Source: PLoS One. 2021 Jul 9;16(7):e0254548. doi: 10.1371/journal.pone.0254548 (PMC8270420; doi:10.1371/journal.pone.0254548)
Supplement: S1 Fig — Kaplan-Meier curve of the time to ADA discontinuation in (A) UC and (B) CD patients within the high drug level group (ADA drug level ≥ 9.2 μg/ml) vs. the low drug level group (ADA drug level < 9.2 μg/ml; p<0.01 by Log Rank). In patients with UC, there was no significant difference in the ADA continuation rate between the high drug level group and the low drug level group (p = 0.19), but there was a significant difference in patients with CD (p<0.01). (PPTX) [file pone.0254548.s001.pptx]

## Slide 1
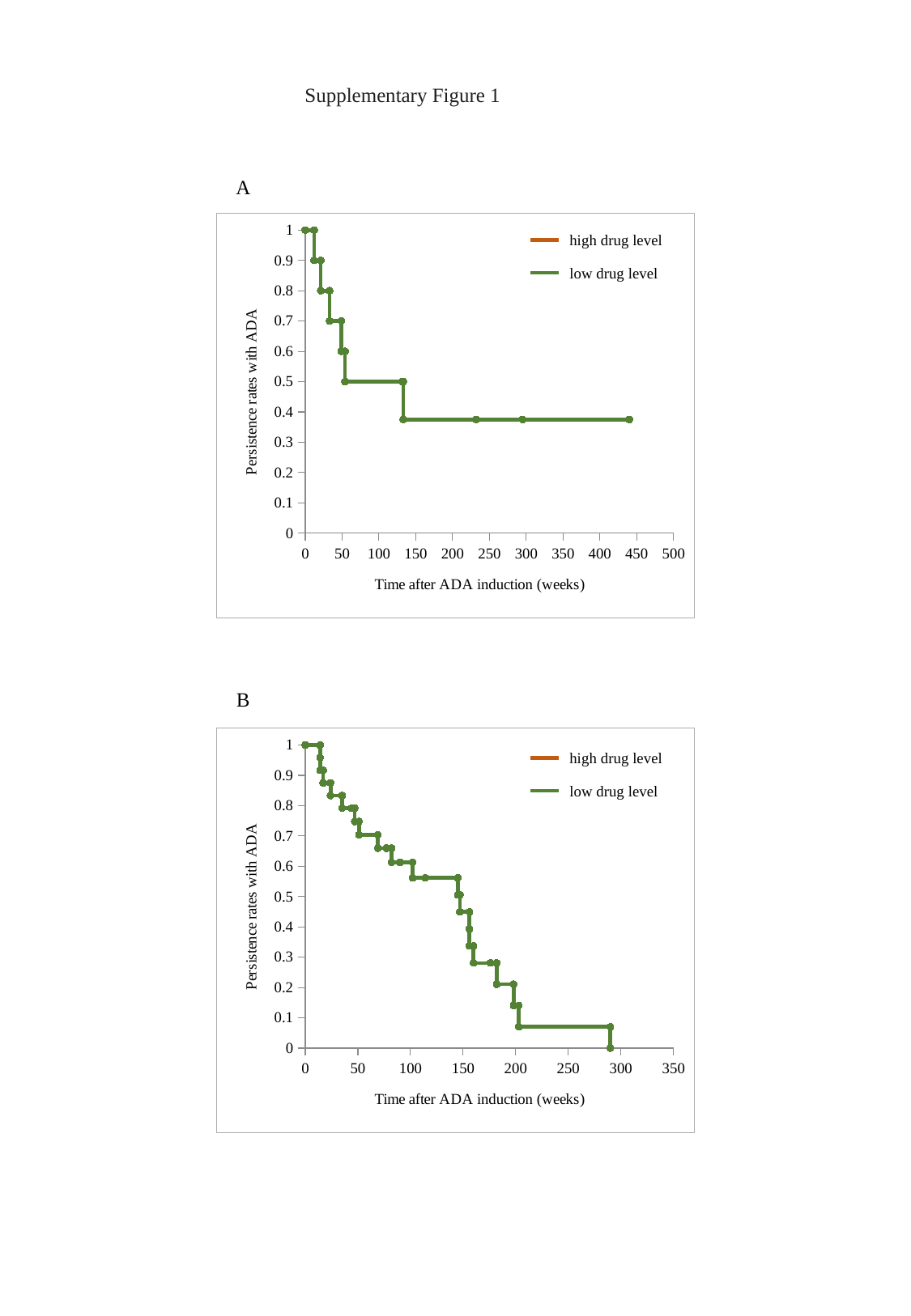

Supplementary Figure 1
A
### Chart
| Category | | |
|---|---|---|high drug level
low drug level
B
### Chart
| Category | | |
|---|---|---|high drug level
low drug level
